# Supplementary material for: A Prospective Observational Study to Investigate Performance of a Chest X-ray Artificial Intelligence Diagnostic Support Tool Across 12 U.S. Hospitals
Source: ArXiv. 2021 Jun 3:arXiv:2106.02118v1. Preprint. [Version 1] (PMC8183017)
Supplement: Supplementary file 1 [file NIHPP2106.02118V1-supplement-1.pdf]

Supplemental Figure 1: Schematic of COVID-19 Diagnostic Model Implementation

Supplemental Figure 2: COVID-19 CXR Diagnostic Model Temporal Validation

Supplemental Figure 3: Distribution of COVID-19 Diagnostic Scores (X-axis) for patients with PCR confirmed positive COVID-19 (purple bars) and non-COVID-19 patients (green bars) during the month of July 2020, prevalence 25.4%

Supplemental Figure 4: External validation COVID-19 Diagnostic AI Scores for COVID-19 positive and negative patients. (a) Box-and-whiskers plot of COVID-19 Diagnostic Scores (y-axis) for non-COVID-19 vs. PCR confirmed COVID-19 from 10,002 CXR from Indiana University. (b) Box-and-whiskers plot of COVID-19 Diagnostic Scores (y-axis) for non-COVID-19 vs. PCR confirmed COVID-19 from 2,002 CXR from Emory University.
